# Less reactogenic whole-cell pertussis vaccine confers protection from *Bordetella pertussis* infection

**DOI:** 10.1128/msphere.00639-24

**Published:** 2025-03-12

**Authors:** Karolína Škopová, Jana Holubová, Barbora Bočková, Eva Slivenecká, João Melo Santos de Barros, Ondřej Staněk, Peter Šebo

**Affiliations:** 1 Institute of Microbiology of the Czech Academy of Sciences, Prague, Czechia; University of Maryland School of Medicine, Baltimore, Maryland, USA

**Keywords:** *Bordetella pertussis*, whooping cough, whole-cell vaccine, reactogenicity, immunogenicity, protection, pertussis toxin, dermonecrotic toxin, lipooligosaccharide

## Abstract

Pertussis resurged over the last decade in most countries that replaced the traditional whole-cell pertussis vaccines (wP) by the less reactogenic acellular pertussis vaccines (aP). The aP vaccines induce a Th2-polarized immune response and by a yet unknown mechanism hamper the clearance of *Bordetella pertussis* from infected nasopharyngeal mucosa. The aP-induced pertussis toxin-neutralizing antibodies effectively prevent the life-threatening pertussis pneumonia in infants, but aP-elicited immunity fails to prevent infection of nasopharyngeal mucosa and transmission of *B. pertussis*. In contrast, the more reactogenic traditional wP vaccines, alike natural infection, elicit a broad antibody response and trigger a Th1/Th17-polarized T cell immunity. We tackled here the reactogenicity of the conventional wP vaccines by genetic modification of the Fim2 and Fim3-producing *B. pertussis* strains used for wP vaccine manufacturing. Mutations were introduced into the genomes of vaccine strains (i) to reduce the TLR4 signaling potency of the lipid A of *B. pertussis* lipooligosaccharide (Δ*lgm*B), (ii) eliminate the enzymatic (immunosuppressive) activity of the pertussis toxin (PtxS1-R9K/E129G), and (iii) ablate the production of the dermonecrotic toxin (Δ*dnt*). Experimental alum-adjuvanted wP vaccines prepared from such triply modified bacteria exhibited a reduced pyrogenicity in rabbits and a reduced systemic toxicity in mice, while conferring a comparable protection from *B. pertussis* infection as the unmodified wP vaccine.

**IMPORTANCE:**

The occasionally severe adverse reactions associated with some lots of the whole-cell pertussis vaccine (wP) led the industrialized nations to switch to the use of less reactogenic acellular pertussis vaccines that confer shorter-lasting protection. This yielded whooping cough resurgence and large whooping cough outbreaks are currently sweeping throughout European countries, calling for the replacement of the pertussis vaccine component of pediatric hexavaccines by an improved wP vaccine. We show that genetic detoxification of the *Bordetella pertussis* bacteria used for wP preparation yields a reduced reactogenicity wP vaccine that exhibits a reduced systemic toxicity in mice and reduced pyrogenicity in rabbits, while retaining high immunogenicity and protective potency in the mouse model of pneumonic infection by *B. pertussis*. This result has now been confirmed in a nonhuman primate model of *B. pertussis* infection of olive baboons, paving the way for the development of the next generation of pertussis vaccines.

## INTRODUCTION

Pertussis, or whooping cough, is a highly contagious respiratory illness caused by *Bordetella pertussis* and less frequently by the human-adapted *B. parapertussis*_hu_ coccobacillli (1). Since the upper airway infection by *B. pertussis* can rapidly progress into a potentially fatal pertussis pneumonia in infants, pertussis used to be the prime cause of infant mortality in developed countries prior to the introduction of whole bacterial cells-based pertussis (wP) vaccines (2, 3). The widespread use of wP yielded a dramatic decrease in whooping cough incidence even if *B. pertussis* continued to circulate to some extent despite vaccine coverage approaching ~86% of the global population (https://www.who.int/teams/immunization-vaccines-and-biologicals/diseases/pertussis). Persisting as the least-controlled vaccine-preventable pediatric infectious disease, pertussis is estimated to still account annually for over 150,000 deaths of the youngest unvaccinated or incompletely vaccinated infants and for dozens of millions of whooping cough cases worldwide (4–7).

The occasional severe adverse reactions due to high endotoxin content in some wP vaccine lots (8–12) led to a decline in public acceptance of the wP vaccines in high-income countries in the 1970s (13, 14). Therefore, less reactogenic acellular pertussis vaccines (aP) were developed in the 1980s, containing only 1–5 purified *B. pertussis* antigens (e.g., chemically or genetically inactivated pertussis toxin [PT], filamentous hemagglutinin [FHA], pertactin [PRN], and serotype 2 and 3 fimbriae [FIM2/3]). Starting from 1998, the aP vaccines progressively replaced the wP vaccine in high-income countries, conferring effective protection from pertussis pneumonia in infants and maintaining pertussis-related mortality very low. However, within a decade from the wP to aP switch, and despite the introduction of booster vaccination, most of the aP-using countries experienced a resurgence of whooping cough outbreaks and a steady increase of the basal inter-outbreak incidence of diagnosed pertussis cases (15–19). It appears, hence, that the immune protection conferred by the aP vaccines wanes rapidly and fails to restrict *B. pertussis* circulation in highly aP-vaccinated populations. In line with that, aP-vaccinated nonhuman primates (*Papio anubis* olive baboons) were not only found to clear a high-dose *B. pertussis* infection from the upper airways notably slower than unvaccinated control animals, but the aP-vaccinated baboons could also effectively transmit *B. pertussis* infection onto both naïve and aP-vaccinated cage mates (20, 21).

Moreover, it has been observed early on that the alum-adjuvanted wP and aP vaccines trigger a strikingly different polarization of T cell immune responses in mice (22–24). Whereas the wP triggers a Th1/Th17-polarized immune response, a Th2 polarization of immune responses was observed in aP-vaccinated mice, baboons, and children (20–28).

Following the resurgence of pertussis in countries that introduced the aP vaccine, the WHO recommended in 2014 that national programs administering wP vaccination should continue to use wP vaccines for primary vaccination series (29). The currently occurring pertussis outbreaks in most highly aP-vaccinated populations of high-income countries raise the question of reformulation of the pediatric hexavaccine and replacement of its aP component by a wP component exhibiting an acceptable reactogenicity profile. Therefore, we examined here if the reactogenicity of the wP vaccine can be reduced by targeted modification of the bacteria used for vaccine manufacturing without compromising the protective potency of the resulting wP vaccine.

## RESULTS

### Genetically detoxified wP vaccine confers high level of protection from lung infection

To construct a wP vaccine exhibiting a reduced reactogenicity (RRwP), we took advantage of low passage stocks of the fully sequenced Fim2 (VS67) and Fim3 (VS377) serotype *B. pertussis* strains that were used in manufacturing of the Czechoslovak DTwP vaccine (30–32). Marker-less modifications were introduced into the chromosomes of the VS67 and VS377 strains by allelic exchange to generate a series of *B. pertussis* strains carrying mutations in virulence genes individually, or in combination. Confirmed mutants were then used to prepare experimental lots of nonadjuvanted wP vaccines that were pre-screened (data not shown) for potential pyrogenicity *in vitro* according to the ICCVAM-Recommended protocol for assessment of potential pyrogenicity of pharmaceutical products based on IL-6 release from MM6 cells (https://ntp.niehs.nih.gov/sites/default/files/iccvam/docs/protocols/pyro-mm6il6.pdf).

The finally retained VS67- and VS377-derived triply modified (3M) strains had each a combination of three modifications that comprised: (i) a Δ*dnt* deletion of the BP3439 gene encoding the neurotropic heat-labile dermonecrotic toxin (33); (ii) a deletion of the BP0398 gene (Δ*lgm*B) required for the glucosyl transferase modification of the phosphate groups of lipid A by glucosamine and thereby for enhancement of the TLR4-activating endotoxic potency of the lipooligosaccharide (LOS) of *B. pertussis* (34–36); and (iii) mutations in the BP3783 gene (*ptx*A) that introduce the R9K and E129G substitutions (R43K and E163G in the unprocessed PtxA) into the PtxA subunit of pertussis toxin, ablating the ADP-ribosylating enzyme activity of its S1 subunit (37, 38). These two 3M strains were next formulated into an experimental RRwP vaccine to evaluate *in vivo* its overall toxicity, pyrogenicity and protective potency in rodents.

**FIG 1:**
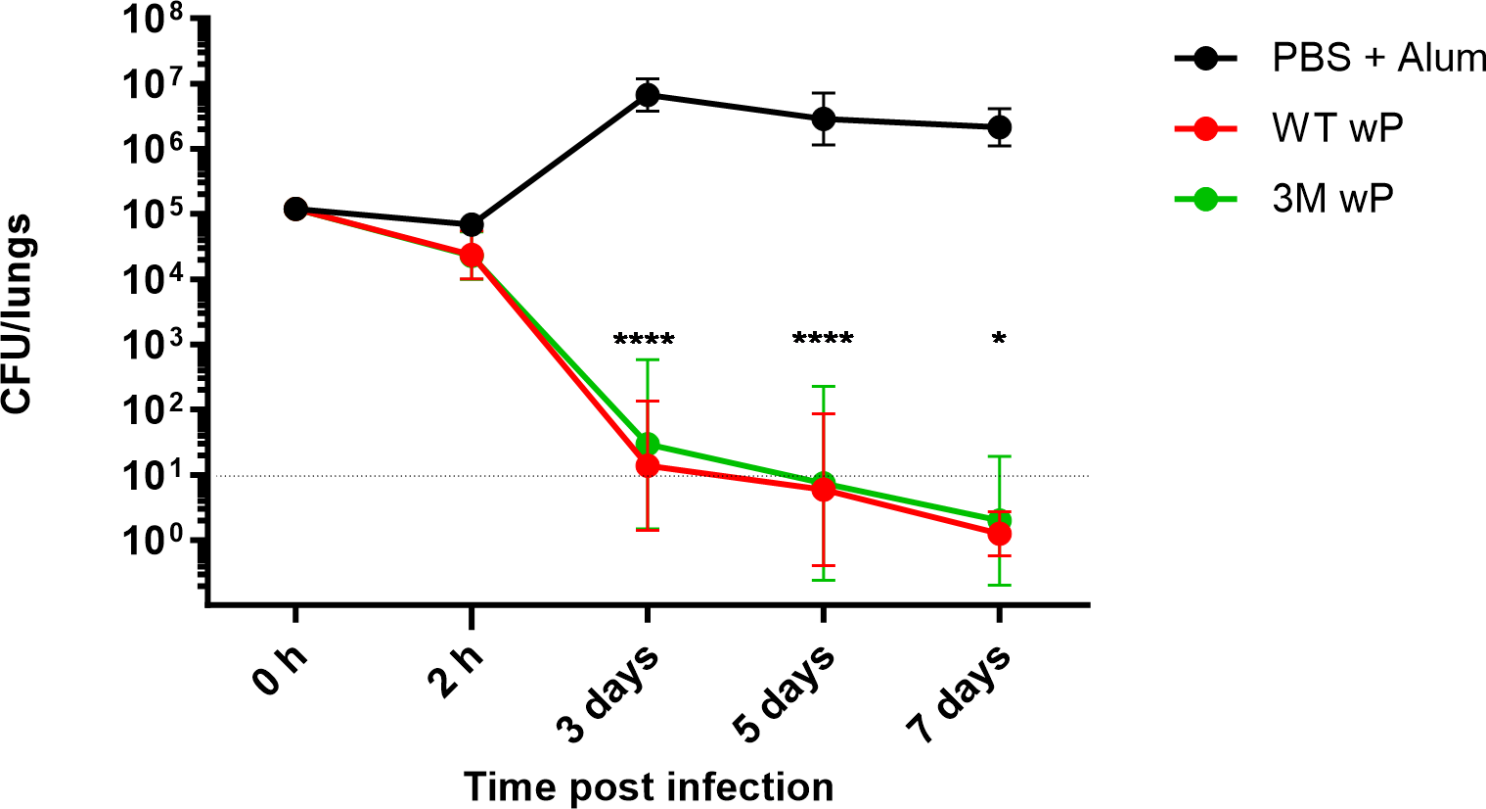
The modified and unmodified wP vaccines confer equal protection from *B. pertussis* infection. Groups of BALB/c mice were immunized intraperitoneally twice at 14-day interval with 1/4 HD of either 3M or WT wP vaccines admixed with alum. Mock-treated control mice received only PBS with alum (0.208% [wt/vol]). Three weeks after the second dose, mice were challenged intranasally with 10^5^ CFU of *B. pertussis* CIP 81.32 (Tohama I) bacteria administered in 50 µL. Mice were euthanized at indicated time points, lungs were aseptically removed, and serial dilutions of lung homogenates were plated on BGA plates for CFU enumeration. Data from three independent experiments with six mice per group and time point were pooled and geometric means of CFU values were calculated. The dashed line indicates the limit of detection (10^1^ CFU). Two-way test ANOVA followed by Sidak´s multiple-comparison test was used to analyze statistical significance between groups. Only significant differences are indicated. *P < 0.05; ****P < 0.0001.

First, the protective antigenicity of wPs made from the modified 3M bacteria was compared to that of wP made from wild-type (WT) VS67 and VS377 bacteria. All strains for experimental wP vaccine formulations were grown under identical conditions and the bacterial cells were inactivated overnight at 37°C with 53.3 mM (0.16% [wt/ol]) formaldehyde. The Fim2 and Fim3-producing bacteria were mixed in a 1:1 ratio into the WT or 3M biomass stocks, which were then formulated with alum (0.62 mg/mL) into the experimental WT or 3M wP (e.g., the RRwP) vaccines. Concentrations of the vaccines were adjusted to 1/4 of human dose (HD) per 0.5 mL in PBS with alum and BALB/c mice were immunized by two consecutive 0.5 mL doses administered intraperitoneally 14 days apart. Three weeks after the second vaccination the animals were infected intranasally with 10^5^ CFU of *B. pertussis* Tohama I bacteria in 50 µL of suspension and bacterial counts in the lungs of infected mice were followed over time. As documented in Fig. 1, in the lungs of naïve mock-immunized mice that received only PBS with alum, the *B. pertussis* counts increased by two orders of magnitude within 3 days post infection. In contrast, mice immunized with 1/4 of HD of either the WT wP or the 3M wP vaccine controlled the lung infection equally effectively within 3 days and cleared the infection by day 7 post challenge. In a two-way comparison, the immunization with the WT and 3M wP vaccines reproducibly conferred an equally high level of protection. Compared to the nonimmunized animals, a drop of bacterial loads by about a half of an order of magnitude was observed in the lungs of WT or 3M wP-vaccinated mice already as early as 2 h after challenge, which was likely due to opsonophagocyting killing of the bacteria. The experimental WT wP and 3M wP vaccines thus exhibited an at least as high protective efficacy in the mouse pneumonic infection model as that reported previously for commercial preparations of wP vaccines formulated into the licensed combination vaccines (39, 40).

### Genetic detoxification importantly reduces toxicity and pyrogenicity of the wP vaccine

**FIG 2:**
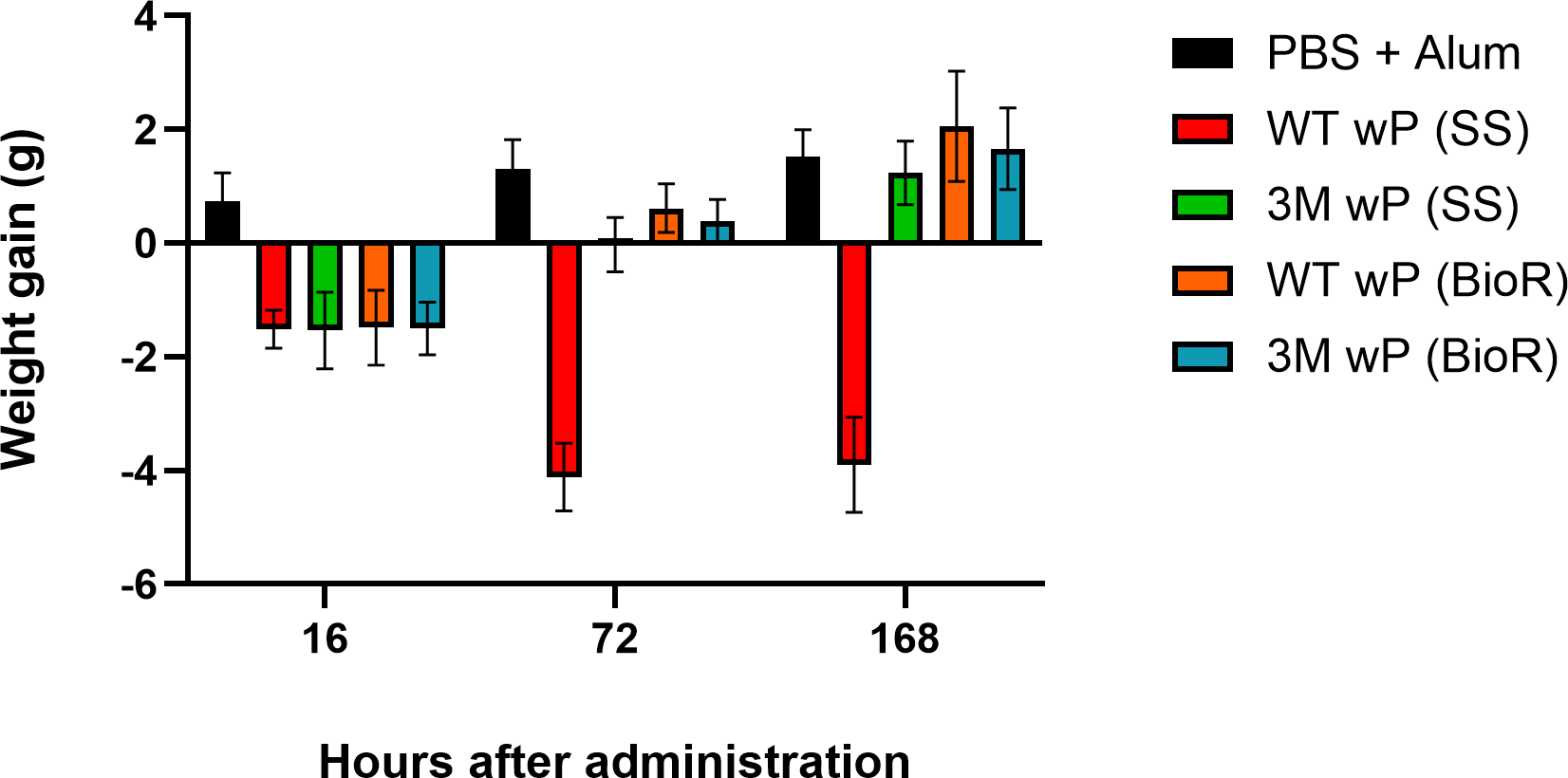
The 3M wP vaccine is less toxic than the WT wP vaccine. Six BALB/c mice per group received intraperitoneally 1/2 HD of the 3M or WT wP vaccine, with mock-treated control mice receiving PBS with alum (0.208% [wt/vol]). Weight gain of mice was determined at indicated time points to monitor vaccine toxicity (WHO Technical report series Annex 6, WHO/IVB/11.11, 2013). Results of mean weight gain from one representative experiment out of three performed is shown.

The experimental WT and 3M vaccines exhibited a striking difference in systemic toxicity that could be readily observed by visual inspection of the health status of immunized animals. Mice that received 1/4 HD of the WT wP vaccine were visibly moribund, had shaggy bristled fur and occasionally succumbed within a day or two after immunization. Such high toxicity of the WT wP vaccine was not unexpected, as the bacteria used for preparation of the experimental wP vaccines were grown in standard Stainer-Scholte media (SS medium) and neither removal of free lipooligosaccharide (LOS), as used in commercial wP vaccine manufacturing, nor heat-inactivation of DNT in the WT wP vaccine was performed. In contrast, the 3M vaccine was clearly much less toxic and did not elicit noticeable morbidity or death of animals immunized with 1/4 HD of the 3M wP vaccine. However, even the 3M wP vaccine prepared from bacteria grown in SS medium did not consistently pass the WHO recommended systemic toxicity test for wP vaccine lot release (WHO technical report series Annex 6, WHO /IVB/11.11, 2013). Therefore, growth medium and culture optimization was undertaken and a buffered Verwey-derived culture medium containing starch and named BioR was used to prepare new experimental WT and 3M wP vaccine lots. As shown in Fig. 2, compared to wP vaccines prepared from bacteria grown in SS medium, the bacteria grown in the BioR medium yielded WT and 3M wP vaccines that were importantly less toxic and passed the mouse weight gain test (MWGT) assay for systemic toxicity. Mice that received 1/2 HD of either the WT wP or 3M wP vaccine lost weight 16 h after immunization but exhibited a positive weight gain at 72 h after vaccine administration and 7 days after immunization gained more than 60% of the mean weight gain of control animals that received PBS only (Fig. 2).

**FIG 3:**
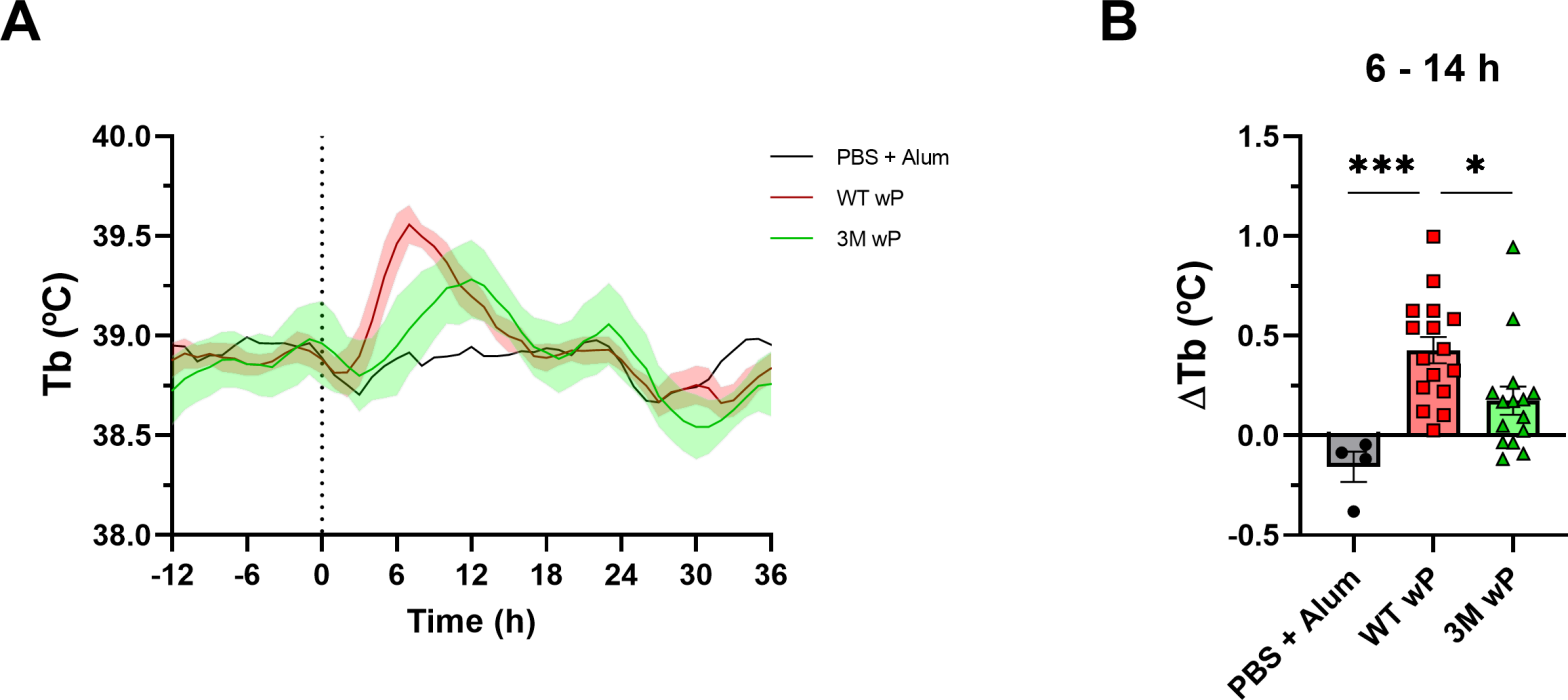
The 3M wP vaccine is less pyrogenic than the WT wP vaccine. (A) The pyrogenicity of the 3M and WT wP vaccines was compared as previously described (41) using female HIL rabbits (2–3 kg) with surgically implanted temperature data loggers (DST micro-T, Star-Oddi, Iceland). Body temperature (Tb) was continuously recorded every 10 min for 3 days prior to vaccination and 3 days after vaccination. On the day of immunization at 8:30 am (dotted vertical line), groups of four rabbits received intramuscularly 1 HD of the WT or 3M wP vaccines in 0.5 mL, or PBS with alum [0.208% (wt/vol) Al(OH)_3_] as a control. Rabbits were euthanized 3 days after immunization, data loggers were extracted, temperature recordings were read and moving average of body temperature in time was calculated for each animal using a window of 4 h moving in 10 min intervals. Data from four experiments with four animals per treatment group were pooled and curves corresponding to mean Tb with error envelopes representing the standard error of the mean (SEM) of the data points are shown (*n* = 16 for WT wP, *n* = 15 for 3M wP and *n* = 4 for PBS + alum). (B) The temperature response (ΔTb) was quantified for each rabbit as the difference between the mean Tb in the 6 to 14 h post-injection interval and the baseline Tb, defined as the mean Tb from −2 to 0 h prior to immunization. Mean ΔTb values were calculated for each rabbit and each treatment group (WT, 3M, and PBS + Alum), with bars representing group means and error bars showing SEM. Statistical analysis was performed using the Sidak`s test to compare ΔTb across groups, with significant differences indicated (*P < 0.05, ***P < 0.001).

Therefore, the pyrogenicity of the WT and 3M wP vaccines prepared from bacteria grown in the BioR medium was assessed in female HIL rabbits with implanted body temperature data loggers (DST micro-T, Star-Oddi) by the method of Kaaijk and colleagues (41). Body temperature was recorded in 10 min intervals with a precision of ±0.1°C for 3 days before and 3 days after intramuscular immunization with 0.5 mL containing 1 HD of the 3M or WT wP vaccine. In total four experiments with groups of four rabbits per vaccine were performed and yielded a clear and statistically significant difference in the pyrogenicity of the two vaccines, as shown in Fig. 3. Considering the circadian oscillation of body temperature of the animals and compared to controls that received only PBS with alum, the administration of the WT wP vaccine provoked on average an increase of the mean body temperature of immunized animals by 0.5°C within 8–10 h from vaccination with a return of the body temperature to the basal level occurring within another 8–14 h on average (Fig. 3A). In contrast, administration of the 3M wP vaccine elicited a notably milder elevation of body temperature by only about 0.25°C on average, exhibiting a slower onset and a faster return of the body temperature to the basal level (Fig. 3A). Hence, the 3M wP vaccine was remarkably less toxic than the WT wP vaccine upon intraperitoneal administration in mice and it was significantly less pyrogenic upon intramuscular administration in rabbits than the vaccine prepared from unmodified bacteria (Fig. 3B).

### The 3M wP vaccine is comparably immunogenic as the unmodified WT wP vaccine

**FIG 4:**
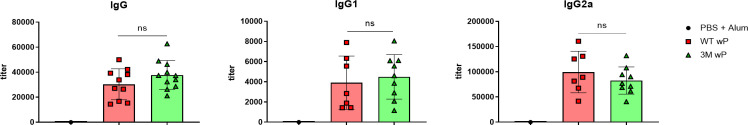
The 3M wP vaccine triggers comparable serum antibody responses as the WT wP vaccine. Mice were immunized with 1/4 HD of the 3M and WT wP vaccine with mock-treated mice receiving PBS with alum [0.208%(wt/vol) Al(OH)_3_] as a control. Sera were collected 3 weeks after the second immunization by retroorbital punction. Titers of *B. pertussis-*specific antibodies in individual sera were determined by whole-cell ELISA on plates coated with heat-inactivated *B. pertussis* CIP 81.32 bacteria (42). Results represent mean antibody titers determined as inflection points of titration curves ± SD. *P* < 0.05.

**FIG 5:**
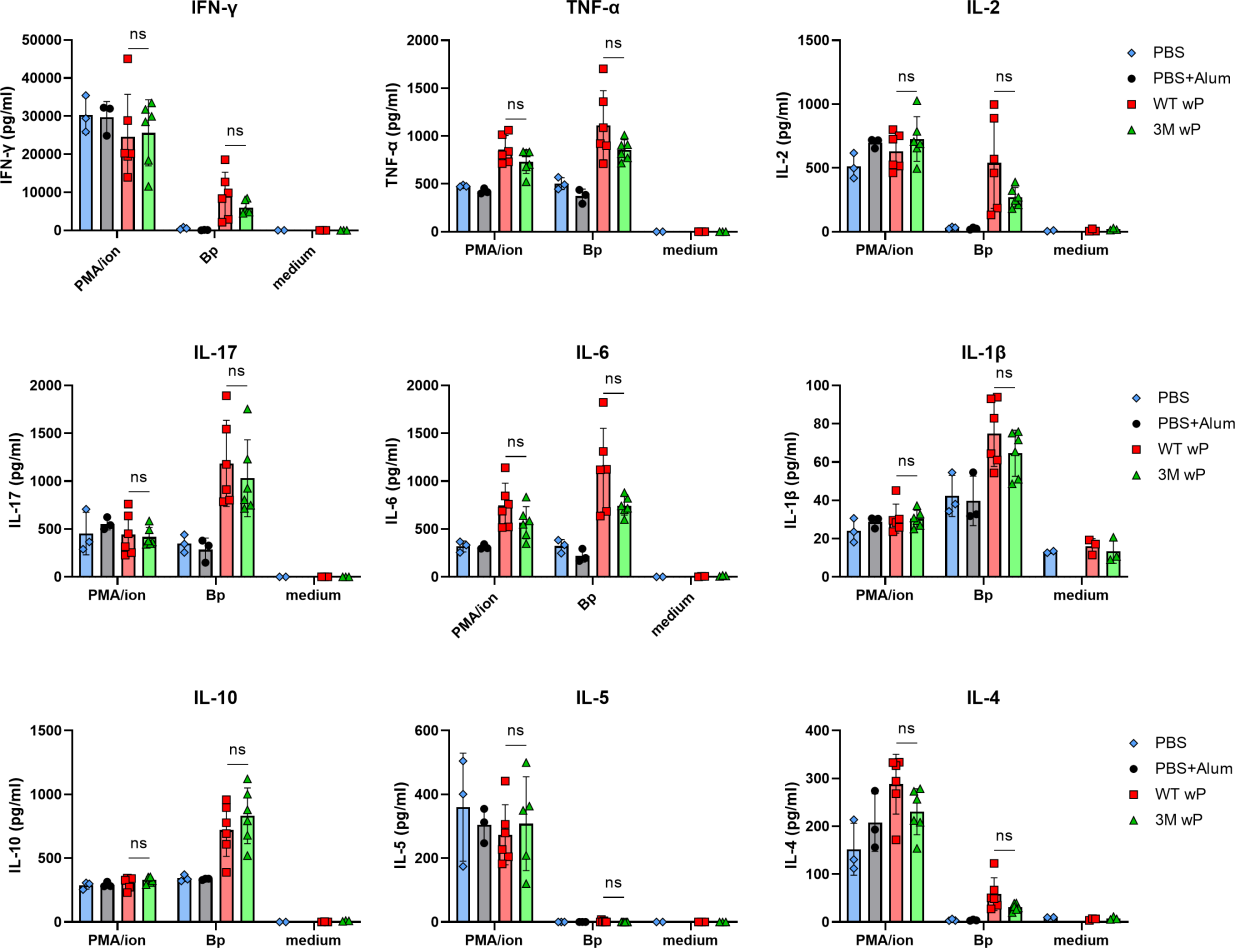
The 3M wP elicits Th1/Th17-polarized immune responses like the WT wP vaccine. Splenocytes of mice immunized as above were collected 3 weeks after the second vaccine dose and restimulated *ex vivo* with heat-inactivated *B. pertussis* cell antigen for 72 h. Sham stimulation with medium and nonspecific polyclonal stimulation with phorbol myristate acetate (PMA) plus ionomycin were used as negative and positive controls, respectively. Supernatants of splenocyte cultures from five mice per group were harvested and cytokine concentrations were determined using specific ELISA kits. Mean cytokine concentrations ± SD are shown. *P* < 0.05.

To compare the immunogenicity of the 3M wP to that of the WT wP vaccine, groups of BALB/c mice were immunized intraperitoneally twice with 1/4 of HD dose of the vaccines, as above, and three weeks after the second dose cytokine responses of splenocytes restimulated with *B. pertussis* antigens and serum antibody levels were determined. As shown in Fig. 4, the WT and 3M wP vaccines induced comparable levels of total *B. pertussis*-specific IgG antibodies, with a very low IgG1 isotype antibody component and a high IgG2a antibody isotype level, suggesting Th1 polarization of the induced immune response (21, 24). Th1 polarization was also clearly apparent from the *in vitro* cytokine responses of splenocytes restimulated with heat-inactivated *B. pertussis* total antigen. Compared to splenocytes from mock-treated mice that received only PBS with alum, used to determine the baseline cytokine levels, the splenocytes from WT or 3M wP-vaccinated mice responded with comparable levels of IL-10 and by very low amounts of the Th2 cytokine IL-4 and no detectable IL-5 (Fig. 5). Splenocytes from both vaccinated animal groups then produced comparably high amounts of the Th1 cytokines IL-1β, IL-6, IFN-γ, and TNF-α and of the IL-17 cytokine (Fig. 5). While the splenocytes from WT vaccinated mice responded with somewhat higher cytokine production than cells from the 3M-vaccinated mice, the differences in the Th1/Th17 cytokine production between the groups were not statistically significant, documenting a high immunogenicity of the 3M wP vaccine.

## DISCUSSION

We report that genetic manipulation of the wP vaccine manufacturing strains of *B. pertussis* yields a significantly less pyrogenic and importantly less toxic wP vaccine (RRwP). Compared to the unmodified wP vaccine, the detoxification of lipid A of LOS, the ablation of the enzymatic activity of pertussis toxin and the deletion of the dermonecrotic toxin gene did not compromise the protective capacity of the vaccine in the mouse lung infection assay. Moreover, this result was corroborated in a parallel study performed in the non-human primate model of olive baboon infection by *B. pertussis* that most truly reproduces the human pertussis pathology (20, 21, 43, 44). For that study, a cGMP batch of the 3M-based modified wP (RRwP) was formulated into an experimental pediatric pentavaccine and proved to be equally efficient in protecting baboon weanlings from high dose intranasal *B. pertussis* infectious challenge as the commercial wP-containing pediatric pentavaccine manufactured by the same process (45). Moreover, the local adverse reactions elicited by the RRwP-containing experimental pentavaccine were importantly milder at a comparable Th17-polarized immune response as that induced by the commercial vaccine (45). These results thus rectify the previously formulated concerns that reduction of the TLR4 signaling potency of the lipid A component of LOS contained in the wP vaccine may compromise the immunogenicity and protective efficacy of the vaccine (46).

Detoxification of the lipid A component of LOS of *B. pertussis* as a path to production of less reactogenic wP vaccine was previously explored by Geurtsen and colleagues (34, 47, 48). Moreover, wP vaccines with reduced levels of endotoxin have previously been successfully produced by extraction of LOS established by the national wP manufacturer in Brazil and such wP vaccine was clinically tested in humans (49, 50). Heat-inactivated suspensions prepared from natural *B. pertussis* isolates lacking the *lgmB* gene-dependent glucosamine modification of lipid A were previously found to exert reduced pyrogenicity in the MM6 cell-based *in vitro* assay and exhibited a reduced capacity to promote dendritic cell maturation, indicating that lack of glucosamine modification of lipid A may impact the potency of the wP vaccine (46). We show that such concerns are not necessarily founded.

We have developed the wP detoxification concept further by removing also the enzymatic (cytotoxic) activity of the pertussis toxin by genetic modification that preserves its protective immunogenicity (37, 38). This modification brings about substantial easing of the vaccine manufacturing process as it eliminates the need for performance of the CHO cell clustering assay used for quantification of the residual pertussis toxin activity in wP vaccine lots prior to their release. Moreover, in line with the ethical 3R requirements for handling of laboratory animals, the ablation of pertussis toxin enzymatic activity also eliminates the need for the performance of leukocytosis promotion test and of the histamine sensitization test, used for *in vivo* assessment of the residual pertussis toxin activity contained in the produced wP vaccine as part of the panel of the lot release assays. These assays alone require the sacrifice of about 100,000 animals annually in the wP manufacturing facilities (51). Hence, no need to perform these assays with the here-described RRwP vaccine represents a significant ethical advance and production cost saving.

Furthermore, the removal of the *dnt* gene from the genome of RRwP vaccine manufacturing strains also eliminates the need for performance of the rather inhumane heat-labile toxin test for residual dermonecrotic toxin activity in the wP vaccine lots that is still performed on large numbers of 4-day-old suckling mice. Therefore, it is worth noting that the here described genetic modifications of the wP manufacturing strains yielding the RRwP vaccine open the way to an importantly more ethical manufacturing of the wP vaccine for human use, significantly reducing the required numbers of laboratory animals to be used in lot release assays, in line with the 3R requirements.

It has previously been established in infectious challenge experiments with wP-vaccinated mice and baboons that the wP vaccine induces a different profile of immune responses and a superior protection of nasopharyngeal mucosa from *B. pertussis* infection than the aP vaccine (21, 52–54). These conclusions have now been confirmed for humans as well. Da Silva Antunes and colleagues (55) showed that primovaccination of humans by the wP vaccine triggers a Th1/Th17-polarized immunity that can be well boosted even by an aP vaccine decades later. In contrast, primovaccination by the aP vaccine triggers a poorly boostable Th2-polarized immunity (21, 52–56). Moreover, the natural infection or parenteral (e.g., i.m or i.p.) vaccination of mice with the wP vaccine was found to induce formation of *B. pertussis*-specific IFN-γ and IL-17-secreting CD44^+^ CD69^+^ CD4^+^-resident memory T cells (T_RM_) that home into airway mucosa and respond to *B. pertussis* infection by secretion of IFN-γ and IL-17 (52–54, 57, 58). These T_RM_ cells were recently found to play a crucial role in the orchestration of early *B. pertussis* clearance from the infected nasopharyngeal mucosa by IL-17-chemoattracted Siglec F^+^ neutrophils (52, 59). In contrast, aP vaccination was found to enable a persistent *B. pertussis* infection of murine nasal cavity by precluding the expansion of protective IFN-γ/IL-17-secreting T_RM_ cells in response to infection (52, 60). These conclusions reached in animal studies are now being validated by the results of aP vaccine booster studies in humans and baboons, revealing that the pediatric aP vaccine-triggered immune polarization towards a Th2 type of immune responses persists for decades from primovaccination (55, 56, 61, 62). Moreover, even repeated high-dose intranasal infection of aP-primed baboons with *B. pertussis* did not trigger expansion of antigen-specific Th1-polarized IFN-γ-secreting CD4^+^ T cells in the aP-vaccinated animals (63). Finally, a recent study by McCarthy and colleagues showed that decades after having been vaccinated as children, the wP-primed humans have importantly higher numbers of pertussis antigen-specific T_RM_ cells in the upper airway mucosa than the aP-primed individuals (62). These observations validate the conclusions reached from animal studies also for aP vaccination of humans. Indeed, epidemiological evidence suggests that aP-elicited immunity confers limited protection from infection of the nasopharynx by *B. pertussis* and enables elevated rates of more-or-less asymptomatic and largely undiagnosed pertussis transmission by highly aP-vaccinated individuals (4, 6, 64, 65).

These recent observations then provide strong support to the hypothesis that the prime cause of pertussis resurgence in countries using the pediatric aP vaccine is the immune mispriming by the aP vaccine towards a Th2-polarized type of immunity. This would yield a delayed clearance of infection, higher levels of *B. pertussis* proliferation in the nasopharynx and more efficient aerosol-mediated transmission of *B. pertussis* infection by the aP-vaccinated compared to wP-primed individuals. Indeed, de Graaf and colleagues recently showed that human volunteers, primed as infants by the wP vaccine and infected as adults intranasally with as much as 10^5^ CFU of *B. pertussis* bacteria, controlled the infection very efficiently (66–68). The infected volunteers did not develop catarrhal pertussis symptoms, did not detectably shed *B. pertussis* bacteria and did not transmit the infection onto close contacts, such as bedroom partners (66–68). These observations collectively support the notion that returning to the use of safe and efficient wP vaccines for immunization of infants would be a way to enhanced control of pertussis in high-income industrialized countries that use the aP vaccine and currently witness massive pertussis outbreaks.

To conclude, we show in an animal model that the pyrogenicity and systemic toxicity of the wP vaccine can be mitigated by genetic modification of the vaccine manufacturing strains without compromising the immunogenicity and protective efficacy of such RRwP vaccine. The here-described RRwP vaccine thus calls for clinical evaluation of pediatric hexavaccines containing a RRwP component with an optimally reduced reactogenicity and preserved protective immunogenicity.

## MATERIALS AND METHODS

### *B. pertussis* strains and growth conditions

The *B. pertussis* Tohama I strain was obtained from the Culture Collection of Institut Pasteur (Paris, France) under the ref. No. CIP 81.32. The Czechoslovak wP vaccine manufacturing strains VS67 (Fim2) and VS377 (Fim3), formulated until 2002 into the DTwP Alditepera vaccine manufactured by the Institute of Sera and Vaccines in Prague, were obtained from Sevapharma a.s. (Prague, Czech Republic). The derived genetically detoxified derivative VS67 3M and VS377 3M strains were generated as described below. To prepare inocula for liquid cultures, *B. pertussis* bacteria were first grown on Bordet-Gengou (BG) agar plates (Difco, USA) supplemented with 1% glycerol and 15% defibrinated sheep blood (LabMediaServis, Jaromer, Czech Republic) at 37°C in a 5% CO_2_ atmosphere for 72 h to visualize hemolysis of isolated colonies. Liquid cultures for mouse infection experiments were obtained by growing *B. pertussis* Tohama I strain CIP 81.32 overnight in modified Stainer–Scholte medium (SSM) (69) supplemented with 3 g/L casamino acids and 1 g/L heptakis-(2,6-di-O-dimethyl)-β-cyclodextrin until mid-exponential phase (*B. pertussis* OD_600_ ~1.0) at 37°C. Liquid cultures for formulation of the experimental wP vaccines were grown in the here-modified Verwey medium, named here BioR, which is buffered with 5 mM HEPES-Na to pH 7 and contains 14 g/L of casamino acids (Difco), 1 g/L of dissolved starch, 1.03 mM KH_2_PO_4_, 2.68 mM KCl, 51 mM NaCl, 1,48 mM MgCl_2_, 2.04 mM CaCl_2_, 1.98 µM FeSO_4_, 57 µM l-cysteine and 0.16 mM nicotinic acid.

### Construction of *B. pertussis* VS67 3M and VS377 3M triple mutant strains

*B. pertussis* VS67 and VS377 triple mutants (3M) were constructed using the allelic exchange vector pSS4245 kindly provided by Dr. Scott Stibitz (70). Briefly, selected fragments of chromosomal DNA of approximately 1400 bp in length were amplified by PCR and inserted into the pSS4245 vector using NotI and BamHI/EcoRI sites, respectively. The construct pSS4245gdPTxS1 (R9K E129G) contained two codon replacements in the *ptx*A gene introducing the R9K and E129G substitutions into the S1 subunit PtxA of pertussis toxin (37, 38). The construct pSS4245Δ*lgm*B contained an in-frame deletion of codons for the residues L3–G539 of the BP0398-encoded LgmB protein (71). The construct pSS4245Δ*dnt*, contained an in-frame deletion of codons for the residues K3–P1463 of the DNT protein (71, 72).

To construct the *B. pertussis* VS67 and VS377 3M strains, the mutations were introduced sequentially into the chromosome by allelic exchange. First, the mutations in the *ptx* gene (R9K E129G) leading to inactive genetically detoxified PT were introduced, then the deletion of the *lgm*B gene leading to an altered lipid A structure was introduced and finally the deletion of the *dnt* gene leading to the absence of DNT. All suicide plasmid constructs used for chromosome mutagenesis were confirmed by DNA sequence analysis prior to use. Following introduction of the mutations into bacterial chromosome by marker-less allelic exchange, the respective portions of the chromosomal DNA were PCR amplified and sequenced to confirm the absence of undesired, and the introduction of the desired mutations/deletions in the *ptx*, *lgm*, and *dnt* loci in the chromosomes of the resulting VS67 and VS377 3M strains. Production of the genetically detoxified pertussis toxoid, of FHA and the absence of DNT production, respectively, were confirmed by western blots (Fig. S1), using specific antibodies for the S1 subunit (PtxA) of PT (mouse polyclonal serum, kind donation from Nicole Guiso, Institut Pasteur, Paris, France), the F1 monoclonal antibody recognizing FHA (kind gift of Camille Locht, Institut Pasteur, Lille, France) and a mouse polyclonal serum against DNT (73). Absence of glucosamine modification of the lipid A of LOS due to deletion of the *lgm*B gene from the chromosomes of the VS67 3M and VS377 3M strains was kindly verified by tandem mass spectrometric analysis of extracted purified LOS by Dr. Alexey Novikov (LPS Biosciences S.A., Orsay, France) as previously described (34–36).

### Whole-cell pertussis vaccine preparation

Experimental wP vaccines were prepared from the *B. pertussis* VS67 and VS377 strains and from the derived genetically modified 3M mutant derivatives. Working seeds were plated on BG agar containing 1% glycerol and 15% defibrinated sheep blood and grown for 4 days at 37°C and 5% CO_2_ before the bacteria were harvested and used to inoculate 20 mL of liquid SS medium supplemented with 3 g/L casamino acids to an OD_600_ = 0.2. Liquid culture inocula were grown for 22 h at 37°C with rotary shaking at 160 rpm until exponential phase and used to inoculate 60 mL of culture medium to OD_600_ = 0.2. The cultures were grown for 22 h at 35°C with shaking (160 rpm) until late exponential phase (OD_600_ = 1.2–1.4). Alternatively, bacterial suspensions for vaccine preparation were grown under the same culture conditions in the BioR medium (defined above). Bacterial cells were chemically inactivated by addition of formaldehyde to 0.16% (wt/vol) with shaking at 37°C for 24 h, followed by 3 days of incubation at 4°C. Inactivated cells were collected and washed three times in phosphate-buffered saline (PBS) using centrifugation at 4.800 x g for 20 min. Vaccine suspensions were formulated with Al(OH)_3_ (as adjuvant, final concentration 0.62 mg/mL) and diluted in PBS to a final concentration of 32 IOU/mL and stored until use at 4°C. Prior to immunization, the vaccines were mixed in a 1:1 ratio (VS67 WT : VS377 WT or VS67 3M : VS377 3M) and the suspensions were further diluted in PBS to the final vaccine concentrations corresponding to 1/4, and 1/2 human dose (HD) per 0.5 mL, respectively.

### Mouse immunization and intranasal infection

Female 5-week-old BALB/c mice (Harlan, The Netherlands) were divided into groups of six animals and housed in cages under specific pathogen-free conditions. Mice were immunized intraperitoneally on day 0 with 500 µL of vaccine (WT wP or 3M wP) containing 1/4 HD (prepared as described above) and boosted 14 days later with the same vaccine. Mice in the control group received 500 µL of PBS containing alum [0.208% (wt/vol) Al(OH)_3_]. Three weeks after the second dose the mice were anesthetized by intraperitoneal (i.p.) injection of ketamine (80 mg/kg) and xylazine (8 mg/kg) in 0.9% saline and inoculated intranasally with a challenge dose of ~1.5 × 10^5^ CFU of *B. pertussis* strain Tohama I (CIP 81.32) in 50 µL delivered in two aliquots of 25 µL per nostril. Six BALB/c mice per group and time point were sacrificed at 2 h and on days 3, 5, and 7 post challenge. Lung tissue was collected aseptically and homogenized in 2 mL PBS with tissue grinder (Heidolph mechanical stirrer RZR 2020, Merck, Darmstadt, Germany). Lung homogenates were serially diluted in PBS, plated onto BG agar and CFUs were enumerated after incubation for 5 days at 37°C in 5% CO_2_ humidified atmosphere. Two-way test ANOVA followed by Sidak’s multiple-comparison test was used to analyze statistical significance between groups. *P < 0.05; ****P < 0.0001.

### Mouse weight gain toxicity test (MWGT)

The combined mouse toxicity test was performed as described (74) following the WHO technical report series Annex 6 (WHO /IVB/11.11, 2013) recommendations. Briefly, 0.5 mL of the WT wP or 3M wP vaccines (1/2 HD), or PBS with alum [0.208% (wt/vol )Al(OH)_3_] used as control, were applied intraperitoneally to female BALB/c mice (14–17 g). Before immunization, mice were randomly divided into groups of 6 animals per cage and weighed individually. The mice were checked daily at 4 pm and weighed again on days 3 and 7 after immunization. The vaccine passed the test if after 72 h from application the total weight of the group was not less than the initial weight and at the end of the seventh day, the mean weight gain of the group was not less than 60% of the mean weight gain of the PBS control group and more than 95% of the animals immunized with the sample survived.

### Pyrogenicity test in rabbits

The impact of vaccine application on body temperature of rabbits was evaluated as described (41) on a contractual basis by Imuna Pharma a.s. (Šarišské Michalany, Slovakia). Body temperature was continuously recorded every 10 minutes using implanted temperature data loggers (DST micro-T, Star-Oddi, Iceland) for 3 days prior vaccination and for 3 days after vaccination according to the Imuna Pharma protocol 545.020-1. Circadian body temperature oscillations were assessed for each rabbit from the recordings over a period of 3 days before immunization. At 8:30 am the groups of four female rabbits (2–3 kg) received subcutaneously (intramuscularly) 1 HD of the WT or 3M wP vaccines in 0.5 mL, or PBS with alum [0.208% (wt/vol) Al(OH)_3_] as control. Rabbits were euthanized 3 days after immunization, data loggers were extracted, and temperature recordings were read. Body temperature data points were used to calculate the moving average body temperature with a window of 4 h moving in 10 min intervals. Four pyrogenicity assessment experiments with groups of four animals per vaccine were performed and body temperature data from 16 animals immunized with the WT wP vaccine and 15 animals immunized with the 3M wP vaccine were pooled to calculate the mean body temperature curves with error envelopes of data points. The temperature response (ΔTb) was quantified for each rabbit as the difference between the mean Tb in the 6–14 h post-injection interval and the baseline Tb, defined as the mean Tb from −2 to 0 h prior to immunization. Mean ΔTb values were calculated for each rabbit and each treatment group (WT, 3M, and PBS + Alum). Two-way test ANOVA followed by Sidak’s multiple-comparison test was used to analyze statistical significance between groups. *P < 0.05; ****P < 0.0001.

### Determination of *B. pertussis*-specific antibodies

Three weeks after application of the second vaccine dose blood of five mice per group was collected from anesthetized animals by retroorbital puncture. Serum was collected at 5,000 × *g* for 10 min at 8°C, stored at −80°C until use and *B. pertussis*-specific antibody levels were determined by whole-cell ELISA as previously described (42). Briefly, ThermoFisher NUNC Maxisorp plates were coated by evaporation of 100 µL per well of *B. pertussis* suspension diluted to OD_600_ of 0.025 in PBS overnight at 37°C without plate lid. The wells were rinsed, blocked with 120 µL per well of 1% BSA in PBS and probed with 1:10 serially diluted sera, starting with a dilution of 1:10 in PBS of control mouse sera and 1:100 dilution for wP-vaccinated mouse sera. After repeated washing with the blocking solution, the bound serum antibodies were detected with a horseradish peroxidase-labelled secondary antibody using OPD and H_2_O_2_ as colorimetric substrate. Plates were read at 492 nm. For titration of total IgG, sheep anti-mouse antibody (GE, clone NA931V) was used at a dilution of 1:3,000. For the titration of IgG1 isotype, a goat anti-mouse antibody (Invitrogen, cat. no. A10551, polyclonal) was used at a dilution of 1:1000 and for titration of the IgG2a isotype antibody, a goat anti-mouse antibody (Abcam, cat. no. ab97245, polyclonal) was used at a dilution of 1:50000. Antibody titers were calculated from the inflection points of the titration curves.

### Cytokine production by restimulated splenocytes

Spleens of five BALB/c mice per group were collected 3 weeks after the second vaccine dose, gently disrupted by passage through a 70 µm strainer. The obtained cellular suspensions were cleared of erythrocytes by treatment with ammonium chloride potassium, washed twice, and cultured in RPMI-1640 medium containing l-glutamine and supplemented with 10% FCS, 1% penicillin, and 1% streptomycin at a cell count of 1 × 10^6^ cells/well in 96-well flat-bottomed tissue culture plates. Splenocytes were stimulated with 1 × 10^7^ heat-killed (30 min, 56°C) *B. pertussis* Tohama I bacteria (MOI 1:10) in a total volume of 200 µL per well. PBS and phorbol myristate acetate plus ionomycin (PMA/ionomycin) (eBiosciences) were used as negative and positive stimulus controls, respectively. Cultures were incubated at 37°C, in 5% CO_2_, and 90% humidity atmosphere for 72 h. Cell viability was assessed before and after incubation by trypan blue exclusion assay. Levels of the IL-1β, IL-2, IL-4, IL-5, IL-6, IL-10, IL-17, TNF-α, and IFN-γ cytokines were determined in the supernatants of stimulated splenocyte suspensions using R&D Systems ELISA kits (Thermo Fisher Scientific) according to the manufacturer’s instructions.

### Statistical analysis

Statistical analysis was performed using the algorithms included in the GraphPad Prism 10 package. Two-way analysis of variance (ANOVA) followed by Sidak’s multiple-comparison tests were used to analyse statistical significance between groups. *P* values of less than 0.05 were considered statistically significant. *P < 0.05; **P < 0.01; ***P < 0.001; ****P < 0.0001.

## Data Availability

Data sets are available at https://doi.org/10.5281/zenodo.14899934. More data will be made available on request.

## SUPPLEMENTAL MATERIAL

Supplemental material msphere.00639-24-s0001.pdf Figure S1.

ASM does not own the copyrights to Supplemental Material that may be linked to, or accessed through, an article. The authors have granted ASM a non-exclusive, world-wide license to publish the Supplemental Material files. Please contact the corresponding author directly for reuse.

## References

[1] Mattoo S, Cherry JD. 2005. Molecular pathogenesis, epidemiology, and clinical manifestations of respiratory infections due to Bordetella pertussis and other Bordetella subspecies. Clin Microbiol Rev 18:326–382. doi:10.1128/CMR.18.2.326-382.2005 15831828 PMC1082800

[2] Gordon JE, Hood RI. 1951. Whooping cough and its epidemiological anomalies. Am J Med Sci 222:333–361. doi:10.1097/00000441-195109000-00011 14877820

[3] Kendrick PL. 1975. Can whooping cough be eradicated? J Infect Dis 132:707–712. doi:10.1093/infdis/132.6.707 1202113

[4] Barkoff A-M, Gröndahl-Yli-Hannuksela K, He Q. 2015. Seroprevalence studies of pertussis: what have we learned from different immunized populations. Pathog Dis 73:ftv050. doi:10.1093/femspd/ftv050 26208655

[5] Frenkel LD. 2021. The global burden of vaccine-preventable infectious diseases in children less than 5 years of age: implications for COVID-19 vaccination. How can we do better? allergy asthma proc 42:378–385. doi:10.2500/aap.2021.42.210065 34474707 PMC8677503

[6] Macina D, Mathur S, Dvaretskaya M, Ekhtiari S, Hayat P, Montmerle M, Daluwatte C. 2023. Estimating the pertussis burden in adolescents and adults in the United States between 2007 and 2019. Hum Vaccin Immunother 19:2208514. doi:10.1080/21645515.2023.2208514 37171153 PMC10184607

[7] Yeung KHT, Duclos P, Nelson EAS, Hutubessy RCW. 2017. An update of the global burden of pertussis in children younger than 5 years: a modelling study. Lancet Infect Dis 17:974–980. doi:10.1016/S1473-3099(17)30390-0 28623146

[8] Baraff LJ, Cody CL, Cherry JD. 1984. DTP-associated reactions: an analysis by injection site, manufacturer, prior reactions, and dose. Pediatrics 73:31–36. doi:10.1542/peds.73.1.31 6606797

[9] Baraff LJ, Cherry JD, Cody CL, Marcy SM, Manclark CR. 1985. DTP vaccine reactions: effect of prior reactions on rate of subsequent reactions. Dev Biol Stand 61:423–428. 3879687

[10] Baraff LJ, Manclark CR, Cherry JD, Christenson P, Marcy SM. 1989. Analyses of adverse reactions to diphtheria and tetanus toxoids and pertussis vaccine by vaccine lot, endotoxin content, pertussis vaccine potency and percentage of mouse weight gain. Pediatr Infect Dis J 8:502–507. doi:10.1097/00006454-198908000-00006 2771530

[11] Baraff LJ, Shields WD, Beckwith L, Strome G, Marcy SM, Cherry JD, Manclark CR. 1988. Infants and children with convulsions and hypotonic-hyporesponsive episodes following diphtheria-tetanus-pertussis immunization: follow-up evaluation. Pediatrics 81:789–794. 3259305

[12] Cody CL, Baraff LJ, Cherry JD, Marcy SM, Manclark CR. 1981. Nature and rates of adverse reactions associated with DTP and DT immunizations in infants and children. Pediatrics 68:650–660. 7031583

[13] Cherry JD, Baraff LJ, Hewlett E. 1989. The past, present, and future of pertussis. the role of adults in epidemiology and future control. West J Med 150:319–328. 2660414 PMC1026455

[14] Melvin JA, Scheller EV, Miller JF, Cotter PA. 2014. Bordetella pertussis pathogenesis: current and future challenges. Nat Rev Microbiol 12:274–288. doi:10.1038/nrmicro3235 24608338 PMC4205565

[15] Chit A, Zivaripiran H, Shin T, Lee JKH, Tomovici A, Macina D, Johnson DR, Decker MD, Wu J. 2018. Acellular pertussis vaccines effectiveness over time: a systematic review, meta-analysis and modeling study. PLoS ONE 13:e0197970. doi:10.1371/journal.pone.0197970 29912887 PMC6005504

[16] Sheridan SL, Ware RS, Grimwood K, Lambert SB. 2012. Number and order of whole cell pertussis vaccines in infancy and disease protection. JAMA 308:454–456. doi:10.1001/jama.2012.6364 22851107

[17] Wilkinson K, Righolt CH, Elliott LJ, Fanella S, Mahmud SM. 2021. Pertussis vaccine effectiveness and duration of protection - A systematic review and meta-analysis. Vaccine (Auckl) 39:3120–3130. doi:10.1016/j.vaccine.2021.04.032 33934917

[18] Witt MA, Arias L, Katz PH, Truong ET, Witt DJ. 2013. Reduced risk of pertussis among persons ever vaccinated with whole cell pertussis vaccine compared to recipients of acellular pertussis vaccines in a large US cohort. Clin Infect Dis 56:1248–1254. doi:10.1093/cid/cit046 23487373

[19] Witt MA, Katz PH, Witt DJ. 2012. Unexpectedly limited durability of immunity following acellular pertussis vaccination in preadolescents in a North American outbreak. Clin Infect Dis 54:1730–1735. doi:10.1093/cid/cis287 22423127

[20] Warfel JM, Merkel TJ. 2014. The baboon model of pertussis: effective use and lessons for pertussis vaccines. Expert Rev Vaccines 13:1241–1252. doi:10.1586/14760584.2014.946016 25182980

[21] Warfel JM, Zimmerman LI, Merkel TJ. 2014. Acellular pertussis vaccines protect against disease but fail to prevent infection and transmission in a nonhuman primate model. Proc Natl Acad Sci U S A 111:787–792. doi:10.1073/pnas.1314688110 24277828 PMC3896208

[22] Mills KH, Barnard A, Watkins J, Redhead K. 1993. Cell-mediated immunity to Bordetella pertussis: role of Th1 cells in bacterial clearance in a murine respiratory infection model. Infect Immun 61:399–410. doi:10.1128/iai.61.2.399-410.1993 8423070 PMC302743

[23] Mills KH, Redhead K. 1993. Cellular immunity in pertussis. J Med Microbiol 39:163–164. doi:10.1099/00222615-39-3-163 8366513

[24] Redhead K, Watkins J, Barnard A, Mills KH. 1993. Effective immunization against Bordetella pertussis respiratory infection in mice is dependent on induction of cell-mediated immunity. Infect Immun 61:3190–3198. doi:10.1128/iai.61.8.3190-3198.1993 8335349 PMC280987

[25] Dirix V, Mielcarek N, Debrie AS, Willery E, Alonso S, Versheure V, Mascart F, Locht C. 2014. Human dendritic cell maturation and cytokine secretion upon stimulation with Bordetella pertussis filamentous haemagglutinin. Microbes Infect 16:562–570. doi:10.1016/j.micinf.2014.04.003 24801497

[26] Dirix V, Verscheure V, Goetghebuer T, Hainaut M, Debrie AS, Locht C, Mascart F. 2009. Monocyte-derived interleukin-10 depresses the Bordetella pertussis- specific gamma interferon response in vaccinated infants. Clin Vaccine Immunol 16:1816–1821. doi:10.1128/CVI.00314-09 19846681 PMC2786394

[27] Dirix V, Verscheure V, Goetghebuer T, Hainaut M, Debrie AS, Locht C, Mascart F. 2009. Cytokine and antibody profiles in 1-year-old children vaccinated with either acellular or whole-cell pertussis vaccine during infancy. Vaccine (Auckl) 27:6042–6047. doi:10.1016/j.vaccine.2009.07.075 19665604

[28] Dirix V, Verscheure V, Vermeulen F, De Schutter I, Goetghebuer T, Locht C, Mascart F. 2012. Both CD4(+) and CD8(+) lymphocytes participate in the IFN-gamma response to filamentous hemagglutinin from Bordetella pertussis in infants, children, and adults. Clin Dev Immunol 2012:795958. doi:10.1155/2012/795958 22550536 PMC3329133

[29] Who. 2016. Pertussis vaccines: WHO position paper, August 2015—Recommendations. Vaccine (Auckl) 34:1423–1425. doi:10.1016/j.vaccine.2015.10.136 26562318

[30] Dienstbier A, Pouchnik D, Wildung M, Amman F, Hofacker IL, Parkhill J, Holubova J, Sebo P, Vecerek B. 2018. Comparative genomics of Czech vaccine strains of Bordetella pertussis. Pathog Dis 76:fty071. doi:10.1093/femspd/fty071 30184175

[31] Pekarek J, Rezabek K. 1959. An endocrinological test for innocuity of the pertussis vaccine. J Hyg Epidemiol Microbiol Immunol 3:79–84. 13654767

[32] Pekarek J, Rezabek K. 1959. The investigation of different components of pertussis vaccine obtained by centrifugation. J Hyg Epidemiol Microbiol Immunol 3:67–78. 13654766

[33] Teruya S, Hiramatsu Y, Nakamura K, Fukui-Miyazaki A, Tsukamoto K, Shinoda N, Motooka D, Nakamura S, Ishigaki K, Shinzawa N, Nishida T, Sugihara F, Maeda Y, Horiguchi Y. 2020. Bordetella dermonecrotic toxin is a neurotropic virulence factor that uses Ca(v)3.1 as the cell surface receptor. mBio 11:e03146-19. doi:10.1128/mBio.03146-19 32209694 PMC7157530

[34] Geurtsen J, Dzieciatkowska M, Steeghs L, Hamstra H-J, Boleij J, Broen K, Akkerman G, El Hassan H, Li J, Richards JC, Tommassen J, van der Ley P. 2009. Identification of A novel lipopolysaccharide core biosynthesis gene cluster in Bordetella pertussis, and influence of core structure and lipid A glucosamine substitution on endotoxic activity. Infect Immun 77:2602–2611. doi:10.1128/IAI.00033-09 19364841 PMC2708539

[35] Marr N, Hajjar AM, Shah NR, Novikov A, Yam CS, Caroff M, Fernandez RC. 2010. Substitution of the Bordetella pertussis lipid A phosphate groups with glucosamine is required for robust NF-kappaB activation and release of proinflammatory cytokines in cells expressing human but not murine Toll-like receptor 4-MD-2-CD14. Infect Immun 78:2060–2069. doi:10.1128/IAI.01346-09 20176798 PMC2863497

[36] Marr N, Tirsoaga A, Blanot D, Fernandez R, Caroff M. 2008. Glucosamine found as a substituent of both phosphate groups in Bordetella lipid A backbones: role of a BvgAS-activated ArnT ortholog. J Bacteriol 190:4281–4290. doi:10.1128/JB.01875-07 18424515 PMC2446747

[37] Nencioni L, Pizza M, Bugnoli M, De Magistris T, Di Tommaso A, Giovannoni F, Manetti R, Marsili I, Matteucci G, Nucci D. 1990. Characterization of genetically inactivated pertussis toxin mutants: candidates for a new vaccine against whooping cough. Infect Immun 58:1308–1315. doi:10.1128/iai.58.5.1308-1315.1990 2323818 PMC258625

[38] Pizza M, Covacci A, Bartoloni A, Perugini M, Nencioni L, De Magistris MT, Villa L, Nucci D, Manetti R, Bugnoli M. 1989. Mutants of pertussis toxin suitable for vaccine development. Science 246:497–500. doi:10.1126/science.2683073 2683073

[39] Godfroid F, Denoël P, de Grave D, Schuerman L, Poolman J. 2004. Diphtheria-tetanus-pertussis (DTP) combination vaccines and evaluation of pertussis immune responses. Int J Med Microbiol 294:269–276. doi:10.1016/j.ijmm.2004.07.007 15532986

[40] Queenan AM, Fernandez J, Shang W, Wiertsema S, van den Dobbelsteen GPJM, Poolman J. 2014. The mouse intranasal challenge model for potency testing of whole-cell pertussis vaccines. Expert Rev Vaccines 13:1265–1270. doi:10.1586/14760584.2014.938642 25029905

[41] Kaaijk P, van der Ark AAJ, van Amerongen G, van den Dobbelsteen GPJM. 2013. Nonclinical vaccine safety evaluation: advantages of continuous temperature monitoring using abdominally implanted data loggers. J Appl Toxicol 33:521–526. doi:10.1002/jat.2720 22407801

[42] van der Ark A, van Straaten-van de Kappelle I, Akkermans A, Hendriksen C, van de Donk H. 1994. Development of pertussis serological potency test. Serological assessment of antibody response induced by whole cell vaccine as an alternative to mouse protection in an intracerebral challenge model. Biologicals 22:233–242. doi:10.1006/biol.1994.1034 7811457

[43] Warfel JM, Beren J, Kelly VK, Lee G, Merkel TJ. 2012. Nonhuman primate model of pertussis. Infect Immun 80:1530–1536. doi:10.1128/IAI.06310-11 22252879 PMC3318410

[44] Warfel JM, Beren J, Merkel TJ. 2012. Airborne transmission of Bordetella pertussis. J Infect Dis 206:902–906. doi:10.1093/infdis/jis443 22807521 PMC3501154

[45] Kapil P, Wang Y, Gregg K, Zimmerman L, Molano D, Maldonado Villeda J, Sebo P, Merkel TJ. 2024. A whole-cell pertussis vaccine engineered to elicit reduced reactogenicity protects baboons against pertussis challenge. mSphere 9:e00647-24. doi:10.1128/msphere.00647-24 39441011 PMC11580402

[46] Brummelman J, Veerman RE, Hamstra HJ, Deuss AJM, Schuijt TJ, Sloots A, Kuipers B, van Els C, van der Ley P, Mooi FR, Han WGH, Pinelli E. 2015. Bordetella pertussis naturally occurring isolates with altered lipooligosaccharide structure fail to fully mature human dendritic cells. Infect Immun 83:227–238. doi:10.1128/IAI.02197-14 25348634 PMC4288873

[47] Geurtsen J, Steeghs L, Hamstra H-J, Ten Hove J, de Haan A, Kuipers B, Tommassen J, van der Ley P. 2006. Expression of the lipopolysaccharide-modifying enzymes PagP and PagL modulates the endotoxic activity of Bordetella pertussis. Infect Immun 74:5574–5585. doi:10.1128/IAI.00834-06 16988232 PMC1594925

[48] Geurtsen J, Vandebriel RJ, Gremmer ER, Kuipers B, Tommassen J, van der Ley P. 2007. Consequences of the expression of lipopolysaccharide-modifying enzymes for the efficacy and reactogenicity of whole-cell pertussis vaccines. Microbes Infect 9:1096–1103. doi:10.1016/j.micinf.2007.04.015 17644385

[49] Dias WO, van der Ark AAJ, Sakauchi MA, Kubrusly FS, Prestes AFRO, Borges MM, Furuyama N, Horton DSPQ, Quintilio W, Antoniazi M, Kuipers B, van der Zeijst BAM, Raw I. 2013. An improved whole cell pertussis vaccine with reduced content of endotoxin. Hum Vaccin Immunother 9:339–348. doi:10.4161/hv.22847 23291935 PMC3859757

[50] Zorzeto TQ, Higashi HG, da Silva MTN, Carniel E de F, Dias WO, Ramalho VD, Mazzola TN, Lima SCBS, Morcillo AM, Stephano MA, Antonio MAR de G, Zanolli M de L, Raw I, Vilela MM dos S. 2009. Immunogenicity of a whole-cell pertussis vaccine with low lipopolysaccharide content in infants. Clin Vaccine Immunol 16:544–550. doi:10.1128/CVI.00339-08 19261771 PMC2668281

[51] Arciniega J, Wagner L, Prymula R, Sebo P, Isbrucker R, Descampe B, Chapsal JM, Costanzo A, Hendriksen C, Hoonaker M, Nelson S, Lidster K, Casey W, Allen D. 2016. Alternatives to HIST for acellular pertussis vaccines: progress and challenges in replacement. Pharmeur Bio Sci Notes 2015:82–96. PMC4980995 27506225

[52] Dubois V, Chatagnon J, Thiriard A, Bauderlique-Le Roy H, Debrie AS, Coutte L, Locht C. 2021. Suppression of mucosal Th17 memory responses by acellular pertussis vaccines enhances nasal Bordetella pertussis carriage. NPJ Vaccines 6:6. doi:10.1038/s41541-020-00270-8 33420041 PMC7794405

[53] Wilk MM, Borkner L, Misiak A, Curham L, Allen AC, Mills KHG. 2019. Immunization with whole cell but not acellular pertussis vaccines primes CD4 TRM cells that sustain protective immunity against nasal colonization with Bordetella pertussis. Emerg Microbes Infect 8:169–185. doi:10.1080/22221751.2018.1564630 30866771 PMC6455184

[54] Wilk MM, Mills KHG. 2018. CD4 T(RM) cells following infection and immunization: implications for more effective vaccine design. Front Immunol 9:1860. doi:10.3389/fimmu.2018.01860 30147701 PMC6095996

[55] da Silva Antunes R, Babor M, Carpenter C, Khalil N, Cortese M, Mentzer AJ, Seumois G, Petro CD, Purcell LA, Vijayanand P, Crotty S, Pulendran B, Peters B, Sette A. 2018. Th1/Th17 polarization persists following whole-cell pertussis vaccination despite repeated acellular boosters. J Clin Invest 128:3853–3865. doi:10.1172/JCI121309 29920186 PMC6118631

[56] Bancroft T, Dillon MBC, da Silva Antunes R, Paul S, Peters B, Crotty S, Lindestam Arlehamn CS, Sette A. 2016. Th1 versus Th2 T cell polarization by whole-cell and acellular childhood pertussis vaccines persists upon re-immunization in adolescence and adulthood. Cell Immunol 304–305:35–43. doi:10.1016/j.cellimm.2016.05.002 PMC4899275 27212461

[57] Dubois V, Locht C. 2021. Mucosal immunization against pertussis: lessons from the past and perspectives. Front Immunol 12:701285. doi:10.3389/fimmu.2021.701285 34211481 PMC8239240

[58] Misiak A, Wilk MM, Raverdeau M, Mills KHG. 2017. IL-17-producing innate and pathogen-specific tissue resident memory γδ T cells expand in the lungs of Bordetella pertussis-infected mice. J Immunol 198:363–374. doi:10.4049/jimmunol.1601024 27864475

[59] Borkner L, Curham LM, Wilk MM, Moran B, Mills KHG. 2021. IL-17 mediates protective immunity against nasal infection with Bordetella pertussis by mobilizing neutrophils, especially Siglec-F^+^ neutrophils. Mucosal Immunol 14:1183–1202. doi:10.1038/s41385-021-00407-5 33976385 PMC8379078

[60] Holubová J, Staněk O, Brázdilová L, Mašín J, Bumba L, Gorringe AR, Alexander F, Šebo P. 2020. Acellular pertussis vaccine inhibits Bordetella pertussis clearance from the nasal mucosa of mice. Vaccines (Basel) 8:695. doi:10.3390/vaccines8040695 33228165 PMC7711433

[61] Gillard J, van Schuppen E, Diavatopoulos DA. 2019. Functional programming of innate immune cells in response to Bordetella pertussis infection and vaccination. Adv Exp Med Biol 1183:53–80. doi:10.1007/5584_2019_404 31432398

[62] McCarthy KN, Hone S, McLoughlin RM, Mills KHG. 2024. IL-17 and IFN-γ-producing respiratory tissue resident memory CD4 T cells persist for decades in adults immunized as children with whole cell pertussis vaccines. J Infect Dis 230:e518–e523. doi:10.1093/infdis/jiae034 38290045 PMC11420794

[63] Kapil P, Wang Y, Zimmerman L, Gaykema M, Merkel TJ. 2024. Repeated Bordetella pertussis infections are required to reprogram acellular pertussis vaccine-primed host responses in the baboon model. J Infect Dis 229:376–383. doi:10.1093/infdis/jiad332 37565807 PMC10873172

[64] Althouse BM, Scarpino SV. 2015. Asymptomatic transmission and the resurgence of Bordetella pertussis. BMC Med 13:146. doi:10.1186/s12916-015-0382-8 26103968 PMC4482312

[65] Domenech de Cellès M, Magpantay FMG, King AA, Rohani P. 2016. The pertussis enigma: reconciling epidemiology, immunology and evolution. Proc Biol Sci 283:20152309. doi:10.1098/rspb.2015.2309 26763701 PMC4721090

[66] de Graaf H, Gbesemete D, Read RC. 2024. Controlled human infection with Bordetella pertussis. Curr Top Microbiol Immunol 445:155–175. doi:10.1007/82_2022_260 36964212

[67] de Graaf H, Ibrahim M, Hill AR, Gbesemete D, Vaughan AT, Gorringe A, Preston A, Buisman AM, Faust SN, Kester KE, Berbers GAM, Diavatopoulos DA, Read RC. 2020. Controlled human infection with Bordetella pertussis induces asymptomatic, immunizing colonization. Clin Infect Dis 71:403–411. doi:10.1093/cid/ciz840 31562530 PMC7353841

[68] Diks AM, de Graaf H, Teodosio C, Groenland RJ, de Mooij B, Ibrahim M, Hill AR, Read RC, van Dongen JJ, Berkowska MA, IMI-2 PERISCOPE Consortium. 2023. Distinct early cellular kinetics in participants protected against colonization upon Bordetella pertussis challenge. J Clin Invest 133:e163121. doi:10.1172/JCI163121 36649086 PMC9974097

[69] Stainer DW, Scholte MJ. 1970. A simple chemically defined medium for the production of phase I Bordetella pertussis. J Gen Microbiol 63:211–220. doi:10.1099/00221287-63-2-211 4324651

[70] Stibitz S. 1994. Use of conditionally counterselectable suicide vectors for allelic exchange. Methods Enzymol 235:458–465. doi:10.1016/0076-6879(94)35161-9 8057916

[71] Holubova J, Stanek O, Juhasz A, Hamidou Soumana I, Makovicky P, Sebo P. 2022. The Fim and FhaB adhesins play a crucial role in nasal cavity infection and Bordetella pertussis transmission in a novel mouse catarrhal infection model. PLoS Pathog 18:e1010402. doi:10.1371/journal.ppat.1010402 35395059 PMC9020735

[72] Kashimoto T, Katahira J, Cornejo WR, Masuda M, Fukuoh A, Matsuzawa T, Ohnishi T, Horiguchi Y. 1999. Identification of functional domains of Bordetella dermonecrotizing toxin. Infect Immun 67:3727–3732. doi:10.1128/IAI.67.8.3727-3732.1999 10417130 PMC96646

[73] Stanek O, Linhartova I, Holubova J, Bumba L, Gardian Z, Malandra A, Bockova B, Teruya S, Horiguchi Y, Osicka R, Sebo P. 2020. Production of highly active recombinant dermonecrotic toxin of Bordetella pertussis. Toxins (Basel) 12:596. doi:10.3390/toxins12090596 32942577 PMC7551409

[74] van Straaten I, Levels L, van der Ark A, Thalen M, Hendriksen C. 2002. Toxicity and immunogenicity of pertussis whole cell vaccine in one animal model. Dev Biol (Basel) 111:47–55. 12678224

