# Supplementary material for: Less reactogenic whole-cell pertussis vaccine confers protection from Bordetella pertussis infection
Source: mSphere. 2025 Mar 12;10(4):e00639-24. doi: 10.1128/msphere.00639-24 (PMC12039235; doi:10.1128/msphere.00639-24)
Supplement: Supplemental material — Figure S1. [file msphere.00639-24-s0001.pdf]

Supplementary Fig. S1.

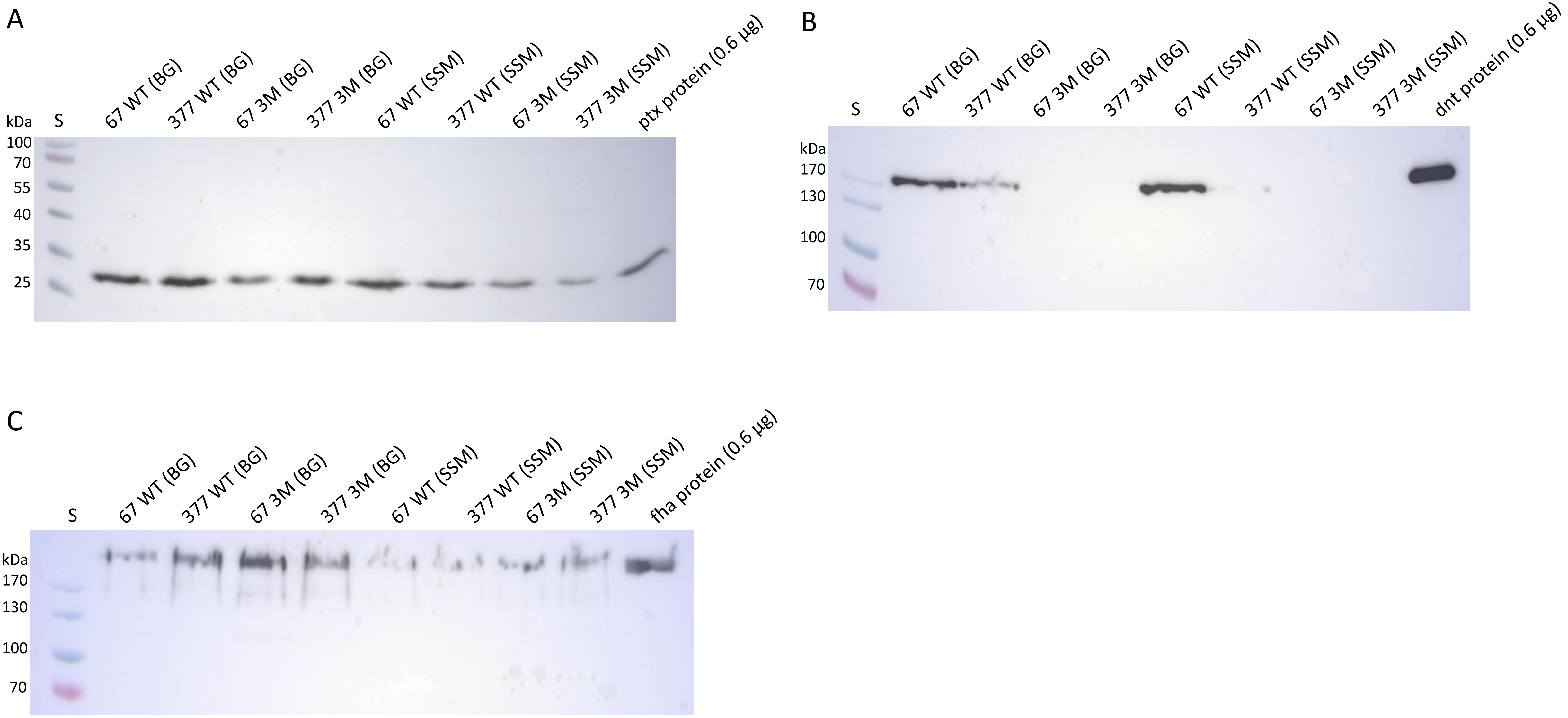

Supl. Fig 1. PT, FHA and DNT production by the modified 3M vaccine strains. *B. pertussis* 67, 377 and its 3M mutant variants were grown on BG agar plates supplemented with blood for 48 h at 37°C (BG) or in liquid SSM medium overnight at 37°C (SSM). Bacterial lysates were prepared and the production of PT, FHA and DNT antigens by the WT wP and 3M wP vaccine strains was probed by Western blots using specific antibodies as described under Materials and Methods. A) Detection of the S1 subunit of PTx. B) Detection of DNT. C) Detection of FHA.
